# Supplementary material for: Learning curve and surgical outcome of robotic assisted colorectal surgery with ERAS program
Source: Sci Rep. 2022 Nov 29;12:20566. doi: 10.1038/s41598-022-24665-w (PMC9709162; doi:10.1038/s41598-022-24665-w)
Supplement: Supplementary file 2 — Supplementary Information 2. [file 41598_2022_24665_MOESM2_ESM.zip › RAL Raw data-Ñ[▒K/058/HS_ñw╝╨░O▒Kñσ.pdf]

正式摘要(電子簽章：000000209740905)

W52 / W52 ☐ 英文標題(注意:無英文姓名-請補病人基本檔!) ☐【讀取】憑證

臺中榮民總醫院

出院病歷摘要

轉入號： 轉出號：

[redacted]

(8)流水編號	
(9)入院日期	110/01/27 (2021) 直腸外科 W52 - 092
(10)轉科日期	
(11)出院日期	110/02/01 (2021) 住院天數計 5 日

診斷

- 入院 :Rectal cancer, 9cm FAV, s/p radiotherapy since 1/19
  hypertension
  type 2 diabete mellitus
  hyperlipidemia
- 出院 :1. Rectal cancer s/p robotic assisted low anterior resection and loop ileostomy on 2021/1/28
  2. hypertension,
  3.type 2 diabete mellitus,
  4. hyperlipidemia

主訴

- bloody stool for about 1 month

病史

- This 75 years old female patient has past history of hypertension, type 2 diabete mellitus, hyperlipidemia. She complained bloody stool for about 1 months. She came to LMD for help. Colonoscopy showed rectal tumor, 9cm from anal verge. Malignancy was proved in pathology report. She was transferred to our hospital. CT showed no obvious distal metastasis. Short course radiotherapy was started from 1/19. This time, she was admitted for surgical treatment.

體檢發現

- PS 70%,
  1100113 No side effect
  - General appearance :
    - easy looking , conscious clear , without cardiopulmonary distress
    - Vital signs自動帶入date 體溫(BT) 脈搏(P) 呼吸(R) 血壓(BP) 身高(BH) 體重(BW) BMI
      20210127 36℃ 91次/分 20次/分 171/98mmHg 143.5cm 57.7kg 28.0
  - HEENT :
    - Eyes: no anemic conjunctiva, no icteric sclera, normal EOM
    - ENT: no otorrhea, no rhinorrhea, no erythematous mucosa, no injected tonsil, no deformity
  - Neck: supple, freely movable, trachea midline,no deformity, no engorged jugular vein , no palpable thyroid or lymph node enlargement
  - Chest:
    - Inspection: Grossly normal, symmetric and fully expansion, no accessory muscle used, no operation scar, no spider angiomata, no gynecomastia
    - Palpation: no palpable mass or nodule, no subcutaneous emphysema, no tenderness
    - Ausculation: clear breathing sound
  - Heart:
    - Percussion: normal heart size
    - Ausculation: regular heart beat, normal S1, S2, no gallop, no murmur
  - Abdomen:
    - Inspection: in
      - flat contour, symetrically, no scar, no stria, no dilated veins, no rashes
    - Ausculation: normoactive bowel sound, no abdominal bruits
    - palpation: soft, no muscle guarding or rigidity, no local tenderness, no rebounding pain, no palpable mass, no palpable pulsation
    - Percussion: no shifting dulllness, no hepatomegaly, no splenomagaly
  -

Peripheral pulsation :	CA	BA	RA	FA	PA	PTA	DPA
Right side :	++	++	++	++	++	++	++/+++
Left side :	++	++	++	++	++	++	++/+++

  - Genito-anus: no tenderness or palpable mass lesion or rectal shelf, normal external genitalia, normal anal tone, brown stool

CRS CONDITION

  - CEA: 7.56 ng/ml
  - Imaging studies(Sono , CT of abdomen and chest Films):
    - 檢驗項目 :CT, PELVIS 報告日期 :1100117
      主 訴 :
      Result: Finding: Imaging Report Form for Colorectal Carcinoma (AJCC 8th) 1. Imaging Date: 20210112 Imaging Modality, Imaging by CT scan Imaging Protocol: Post IV contrast CT scan of the whole abdomen with 600cc oral contrast. 2. Tumor location / Size Location: Rectum Size: Measurable: 2.4 cm (largest diameter) 3. Tumor invasion Tumor cannot be visualized in this imaging study 4. Regional nodal metastasis No 5. Distant metastasis (In this study) No or Equivocal 6. Other findings: (1) Presence of segmental wall thickening of rectum(cor#37), about 2.4cm, rectal cancer may be considered. (2) Several low attenuation lesions in bilateral lobes of liver, up to 1.0cm, smal cysts are considered first. Suggest follow-up. (3) A 0.7cm gallstone is noted. (4) A 1.5cm diverticulum arising from 2nd portion of duodenum. (5) Slight hypertrophic change of bilateral adrenal glands. (6) A 1.2cm nodule at LUQ abdomen, abutting to spleen, in favor of accessory spleen. (7) No significant retroperitoneal lymphadenopathy. (8) Bil. visible lower lungs are clear. (9) DJD change of T and L spine. (10) Calcifications of coronary arteries, R/I CAD.
      Impression: R/I rectal cancer. No significant regional lymphadenopathy or intra-abdominal metastasis.
  -
    - Lung

手術日期及方法

- 手術日期及方法20210128 1. Robotic (XI) assisted low anterior resection + loop ileostomy
  2. Intraoperative colonoscopy : (-).
  3. High ligation (+) : At IMA root. Left colic artery and IMV were also ligated via robotic endoclips.
  4. Take down from the splenic flexure : (-)
  5. Robotic endoGIA (45-3.8) X2 + EEA (29mm) + OpenGIA (80-4.8) X1 were applied to completed side to end anastomosis
  6. Proximal cut end: 8 cm, distal cut end: 3 cm
  7. ADHESION BARRIER FILM was placed below the central abdominal wound

住院治療經過

- After admission ,pre-o survey was done and showed no contraindications, therefore operation was done on 1/28. After ther operation, the patient tried liquid diet well, CVC was removed on 1/30, under stable condition, the patient was discharge with OPD follow up.

合併症

一般檢查

特殊檢查

病理報告

其它

出院時情況

- 3.改門診治療

出院指示

-

藥名	劑量	劑量單位	用法	頻次	數量	數量單位	現況	天數	單位
Voren-K tab 25mg	1	TAB	PO	TID	9	TAB	TH	3	天
Xanax tab 0.5mg	1	TAB	PO	HSPRN	8	TAB	TH	8	天
(□)Takepron OD tab 30mg	1	TAB	PO	QDAC	8	TAB	TH	8	天
Ultracet tab	1	TAB	PO	BIDPRN	16	TAB	TH	8	天
Vesicare tab 5mg	1	TAB	PO	QD	8	TAB	TH	8	天

出院計劃

主治醫師_2062F 蔣鋒帆_____ / 住院醫師_2332G 林志安_____

正式摘要(電子簽章：**000000209740905)**

W52 / W52

doctorRank:B
